# RETRACTION: Crystal Growth and Kinetic Behaviour of *Pseudoalteromonas espejiana* Assisted Biosynthesized Gold Nanoparticles

**DOI:** 10.1155/omcl/9761051

**Published:** 2025-10-06

**Authors:** Oxidative Medicine and Cellular Longevity

RETRACTION: R. Gupta, G. Kumar, S. S. Das, et al., “Crystal Growth and Kinetic Behaviour of *Pseudoalteromonas espejiana* Assisted Biosynthesized Gold Nanoparticles,” *Oxidative Medicine and Cellular Longevity* 2020 (2020): 6501294, https://doi.org/10.1155/2020/6501294.

The above article, published online on 22 July 2020 in Wiley Online Library (wileyonlinelibrary.com), has been retracted by agreement between the journal Editor-in-Chief, Jeannette Vasquez-Vivar, and John Wiley & Sons Ltd. The retraction has been agreed due to similarities in the data presented in this article and the thesis of Gupta [1], despite being on different species.

Concerns have also been raised in relation to the COI and funding statements, as well as for the permissions obtained to use the microbial sample.

The authors provided a response to the concerns, but could not provide the raw data for the article. After assessment by the Chief Editor, the response was not deemed satisfactory and the article is retracted.

The authors agree with the retraction.
